# Community-Driven Grassroots Intervention on Adolescent Vaping Attitudes, Harm Perceptions, and Knowledge: Randomized Controlled Trial

**DOI:** 10.3390/ijerph23060789

**Published:** 2026-06-11

**Authors:** Mirza Ali Anser Beg, Yahya Dawood, Scott Burton Patten

**Affiliations:** 1 Cumming School of Medicine, University of Calgary, Calgary, AB T2N 4N1, Canada; mirza.beg2@ucalgary.ca; 2 Department of Community Health Sciences, University of Calgary, Calgary, AB T2N 4N1, Canada; patten@ucalgary.ca

**Keywords:** vaping, adolescents, grassroots intervention, e-cigarettes, public health, youth engagement, school-based

## Abstract

**Highlights:**

**Public health relevance—How does this work relate to a public health issue?**

- Adolescent vaping is a significant and rising public health concern in Canada, where roughly 13% of youth aged 15 to 19 report past-30-day e-cigarette use, yet conventional school-based prevention programs have shown limited effectiveness.
- This study evaluates a community-driven, youth-developed video as a novel alternative to traditional expert-led vaping education for early adolescents.

**Public health significance—Why is this work of significance to public health?**

- To our knowledge, this is one of the first randomized controlled trials to test whether a grassroots, youth-led intervention can outperform an expert-developed comparator, and it produced a significantly greater shift in adolescents’ e-cigarette harm perceptions (medium effect size) alongside higher ratings for enjoyment and perceived knowledge gain.
- The findings indicate that involving youth in the design of prevention messaging can enhance relatability and impact, while also identifying an important caution, namely that such messaging may overstate the harm of e-cigarettes relative to combustible cigarettes.

**Public health implications—What are the key implications or messages for practitioners, policy makers and/or researchers in public health?**

- Schools and public health agencies may benefit from partnering with youth councils and community groups to co-develop prevention materials, treating grassroots initiatives as complements to traditional strategies.
- Future youth-led interventions should preserve their engagement advantages while ensuring balanced relative-risk communication, and should be evaluated through multi-site, longitudinal trials with behavioral endpoints.

**Abstract:**

This study evaluated the effectiveness of a youth-developed vaping intervention created by the Airdrie Board of Youth Affairs (ABYA) in changing Grade 7 and 8 students’ knowledge, attitudes, and harm perceptions regarding e-cigarette use. The vaping video trial followed a preceding local education program on smoking harms. A total of 107 students were randomly assigned to either the intervention group (ABYA video) or the control group (expert-developed video). Four instruments were used: the Video Survey, Personal Information Questionnaire, Knowledge and Attitudes Regarding E-cigarette Ingredients, Safety, and Addictive Properties (KAS), and the E-cigarette Harm Perception and Reduction (EHI). Pre- and post-intervention data were analyzed using *t*-tests and Mann–Whitney U tests. Compared with controls, the intervention group showed a significantly larger mean decrease in total EHI scores (mean change 9.05 vs. 2.06; t(105) = 3.34, *p* = 0.001; Cohen’s d = 0.65), indicating that the youth-developed video increased the perceived risk of vaping relative to cigarettes to a greater extent than the expert-developed video. Students also rated the ABYA video significantly higher on a 5-point scale for overall enjoyment (3.13 vs. 2.33; *p* < 0.001; Cohen’s d = 0.81) and for perceived increase in knowledge about vaping (3.34 vs. 2.84; *p* = 0.023; Cohen’s d = 0.44). The KAS instrument showed low internal consistency in this sample, so item-level KAS findings were treated as exploratory. Notably, unlike the expert-developed video, which explicitly acknowledged uncertainty, the ABYA video conveyed a clearer and more direct message, which may have contributed to its greater appeal while also carrying a risk of overstating absolute risk of e-cigarettes relative to cigarettes. Overall, these findings suggest that grassroots, youth-led interventions may be an effective approach for adolescent vaping education and may offer advantages over traditional expert-developed messaging in some contexts.

## 1. Introduction

Vaping refers to the act of inhaling and exhaling aerosol produced by electronic cigarettes (e-cigarettes) or similar devices [1]. Vaping is generally considered less harmful than smoking traditional combustible tobacco cigarettes and has been used as a tool to support tobacco smoking cessation [2]. However, clinical manifestations of vaping-related lung injury have been observed, and vaping is associated with a range of serious health hazards including nicotine addiction, lung damage, respiratory issues, and cardiovascular problems [1,3,4]. In Canada, approximately 13% of youth aged 15–19 and 12% of those aged 20–24 have vaped in the past 30 days, compared to only 3% of Canadians over 25 [5]. Vaping has thus presented itself as a significant public health concern, especially among youth and adolescents in Canada [5]. E-cigarette companies employ targeted marketing strategies that make vaping products attractive and appealing to young individuals [6,7]. The ongoing brain development during adolescence increases susceptibility to nicotine addiction, potentially leading to long-lasting cognitive consequences and a higher risk of substance-use disorders [8]. While e-cigarettes are generally considered less harmful than combustible cigarettes [9], they still carry health risks and can produce nicotine dependence, particularly among youth with research showing that in certain health outcomes both e-cigarettes and cigarettes have similar impacts [10,11].

General ignorance and targeted marketing surrounding vaping is a pervasive issue, contributing to its uptake among youth [12]. Misinformation may downplay the risks associated with vaping and be partly responsible for the recent youth vaping epidemic [13,14,15]. Increasing perceived risk, making these risk perceptions more accurate, and lowering misinformation regarding vaping can reduce youth vaping-associated harm [15,16]. Therefore, effective public health interventions must be developed for youth to increase perceived risk and lower misinformation [12,17]. Interventions observed have primarily been public education and school-based efforts, along with community-based interventions, healthcare provider interventions, aerosol-free policies, age restrictions, and advertising and promotion restrictions [18]. However, Williams et al. found that current school-based e-cigarette prevention programs are insufficient in preventing e-cigarette initiation and called for enhanced support and resources [19]. Another study by Struik et al. evaluated vaping intervention programs across North America and found that many programs do not fully leverage evidence-based behavior change methods, suggesting a need for more comprehensive and theoretically grounded approaches to effectively reduce vaping among youth [20]. Together, these findings emphasize the importance of updating vaping prevention strategies to reflect current trends, ensure stronger engagement, and address gaps in effectiveness. Recent systematic reviews highlight this urgency; one meta-analysis of school-based programs in 2024 found that while short-term improvements in knowledge, attitudes, and intentions were achieved, these interventions failed to affect long-term e-cigarette use [21]. Another 2025 review of health messaging strategies highlights that digital, behaviorally informed approaches, such as targeted messaging campaigns, are emerging as more effective in altering youth vaping perceptions and behaviors [22]. Importantly, a systematic review on youth vaping in the digital age highlighted a significant research gap in youth-specific e-cigarette interventions, despite the increasing prevalence and associated risks [22].

Grassroots interventions are community-driven efforts typically initiated and led by volunteering community members and are based on principles of active community engagement in creating solutions [23]. These interventions leverage local knowledge, experiences, and resources to develop and implement solutions tailored to the community’s specific needs and contexts [23]. Youth engagement is a crucial determinant of success in public health interventions, yet it has often been overlooked [24,25]. This oversight is significant as involving youth in the creation and implementation of interventions ensures that related strategies are relevant, relatable, and more likely to be effective [24,25]. Engaging youth not only empowers them but also enhances the credibility and acceptance of the interventions among their peers [24,25]. Thus, developing youth-driven initiatives can lead to more impactful and sustainable outcomes in addressing vaping among adolescents [24,25]. This approach is grounded in community-based participatory research and asset-based community development, both of which emphasize lay leadership, local ownership of problem definition, and the use of insider knowledge in shaping solutions. The grassroots model is distinct from peer-led and school-based programs in an important respect: peer-led programs typically have adolescents deliver content that was designed by adults, whereas a grassroots intervention has community members, in this case youth, generate the content itself. The present study addresses a gap in the prevention literature by directly testing whether a vaping intervention conceptualized, scripted, and produced by adolescents can outperform an expert-developed comparator on adolescent knowledge, attitudes, and harm perceptions.

To date, prevention efforts have followed a more traditional approach whereby interventions are designed and implemented by organizations or institutions external to the community, such as government agencies, public health organizations, or academic institutions. These interventions often follow a top-down approach, relying on standardized methods and expert knowledge to address public health issues. While traditional interventions benefit from established research and professional expertise, they may lack the personalized touch and contextual sensitivity of grassroots and youth-led efforts [23].

The Airdrie Board of Youth Affairs (ABYA) is a youth council formed to empower youth in Airdrie to deal with issues that affect them, one of which they identified as vaping. They subsequently developed a community-based informational video to educate fellow youth on the risks and dangers of vaping. This grassroots vaping intervention was implemented in a local context already shaped by Alberta’s broader provincial tobacco-prevention efforts, including Alberta Health Services’ Academy for Tobacco Prevention, a province-wide school-based smoking prevention program launched in 2016 for students in Grades 4 to 6, within a broader tobacco reduction strategy that in Alberta dates back to 2002. Therefore, the intervention developed by ABYA focused on vaping over cigarette use as students were already being exposed to education regarding the latter. This study was designed to explicitly test the hypothesis that grassroots, youth-led intervention (ABYA video) would be more effective than a traditional expert-developed intervention (NIH video) in improving adolescents’ knowledge, attitudes, and harm perceptions related to e-cigarette use. We also sought to determine whether students would evaluate a youth-developed intervention more favorably than a traditionally developed one.

## 2. Materials and Methods

### 2.1. Ethics

This study was conducted according to the Tri-Council Policy Statement 2022 and international standards of Good Clinical Practice for all studies. University of Calgary research policies and procedures were also followed. The study received ethical approval from the University of Calgary Conjoint Health Research Ethics Board ID: REB23-1437. Approval was also granted by the Rocky View Division Research Committee to obtain permission to conduct research at Muriel Clayton Middle School.

### 2.2. Sample Characteristics

The population studied included Grade 7 and 8 children attending the Muriel Clayton Middle School in Airdrie, Alberta, Canada. The intervention targeted Grade 7 and 8 students between the ages of 12 and 16. Teachers identified and helped to exclude from analysis those with significant language barriers or developmental delays, although these students still participated during the intervention to ensure an inclusive environment. Muriel Clayton Middle School was selected as the study site because of an existing collaborative relationship between the school administration and the ABYA, which had previously identified vaping as a priority concern within the school community. This established partnership allowed the youth-developed content to be delivered without modification, preserving fidelity to the grassroots model. A single-site design was therefore adopted on grounds of feasibility, access, and intervention fidelity, with the corresponding trade-off in external validity addressed in the Limitations section.

### 2.3. Intervention

The intervention was developed using a Community-Based Participatory Research approach where the ABYA conceptualized, designed, and implemented the intervention themselves with external input from community partners and informal interactions with public health researchers. The ABYA is a group of 10 members between the ages of 12 and 22 years. The intervention is in a video format and can be found in the Supplementary Materials along with the control video. Key topics discussed in the video were nicotine addiction and brain development, chemical exposures in vapes, nicotine content comparisons with cigarettes, social/behavioral risks, and refusal skills and resources. The ABYA intervention video featured youth of similar age to the target audience explaining various facts related to vaping through visual demonstrations and was 3 min 48 s in length. The ABYA video was youth-created, humorous, and used vivid risk framing, which may increase engagement but can trade off against balanced relative-risk communication. The comparison video, of similar content and length, was produced by the NIH and was publicly available on YouTube. The NIH video ultimately represented the traditional method of developing substance abuse interventions for youth (i.e., not grassroots-led where the target audience, in this case youth, lead the conceptualization, designing, and implementation of the intervention).

In contrast, the comparison video, of similar content and length, was produced by the National Institutes of Health (NIH) and represented the traditional expert-driven model of health education. Unlike the ABYA video, which was co-created and narrated by peers, the NIH video was developed by researchers and health professionals with little direct input from adolescents. While both videos conveyed information on nicotine addiction, health risks, and prevention, the ABYA video uniquely emphasized peer-to-peer relatability, using language, tone, and presentation styles tailored by youth themselves. This grassroots design process is the major strength of the ABYA intervention, as it leveraged the perspectives of adolescents to produce content that was more engaging, credible, and relatable for the target audience.

### 2.4. Questionnaires

Four data collection tools were used for the purposes of this study: The Video Survey; Personal Information Questionnaire; Knowledge and Attitudes Regarding E-cigarette Ingredients, Safety, and Addictive Properties survey (KAS); and The E-cigarette Harm Perception and Reduction survey (EHI).

The Video Survey, adapted from a previous study [26], assessed students’ views on the intervention’s impact on their vaping knowledge and attitudes. It included questions on prior vaping experience and short-answer opinions on the intervention’s effectiveness.

The Personal Information Questionnaire collected demographic information: age, gender, and grade. No other identifying personal information was collected (name, contact information, etc.) and all results were anonymous.

The adapted KAS survey, a validated 10-item questionnaire, used a 4-point Likert scale (Strongly Agree to Strongly Disagree) to assess knowledge and attitudes [16]. This survey was adapted from a previously published study and was developed from qualitative interviews and pilot studies done with youth [16].

The validated E-cigarette Harm Perception and Reduction (EHI) survey was also adopted and has 14 items, utilizing a 7-point rating scale for responses, spanning from “strongly disagree” to “strongly agree” [18]. These items were organized into three categories that reflected beliefs about e-cigarettes: their reduced harm compared to cigarettes (for example, “E-cigarettes offer a safer nicotine intake method”; comprising 7 items, with a reliability coefficient α of 0.92 in the present dataset), their potential to enhance the health of smokers (for example, “E-cigarettes can improve breathing and lessen coughing”; consisting of 3 items, with α = 0.82), and their effectiveness as a cessation tool (for example, “E-cigarettes assist individuals in quitting smoking”; including 4 items, with α = 0.85). Collectively, these 14 items demonstrated strong internal consistency (α = 0.94). For this analysis, scores from these items were used to construct a “harm perceptions” latent variable, which was defined by the combined indices of the three belief factors.

### 2.5. Study Procedure

Students in Grade 7 and 8 from the Muriel Clayton Middle School were recruited to take part in the study and were sent home with parental consent and study information forms 1 week prior to the intervention. Inclusion criteria required that students (1) were enrolled in Grade 7 or 8 at Muriel Clayton Middle School, (2) had parental consent to participate, and (3) were able to read and understand English at a functional level sufficient to complete the questionnaires. Each classroom was evenly divided into groups A and B. Teachers used randomization of class lists in Excel (=RANDBETWEEN(1, 2)) to produce a random list of students in groups A or B, where 1 corresponded with A and 2 with B. There was no gender or racial bias in the selection of participants. After all students were assigned, they proceeded to their groups’ assigned room and received an envelope containing the surveys. The envelopes were labeled with the student’s study ID and started with the letter A or B corresponding to their assigned group (for example, A68 would indicate they are number 68 belonging to group A). Group A was the experimental group receiving the ABYA video and Group B was the control group receiving the NIH video.

Classroom teachers were given a script that they used to introduce the study to the students. This pre-intervention script was identical for both groups. Students were informed that their responses would be completely anonymized to minimize the risk of social desirability bias. School teachers were consulted to identify any students who did not meet the inclusion criteria. Each student participating in the study was given an envelope containing a unique study ID and 2 booklets. The first booklet contained the Personal Information Questionnaire, one copy of the KAS and EHI surveys, and an informed consent form describing the study. The students’ decision to return the envelope was an indication of implied assent. The other booklet contained 2 more copies of both KAS and EHI surveys and the Video Survey. The students completed the former booklet with the Personal Information Questionnaire before watching any video, and the latter booklet with Video Survey after watching the video assigned to their group. Individuals present in the room included one research assistant allowed to walk around the class to address any concerns students had, and one school staff member who was asked to remain at their desk to avoid biasing student responses. Once all the forms were completed, students were asked to place everything back into the envelopes. After completing the assigned video and associated evaluations, students were shown the video they had not seen yet to ensure that no student was excluded from any of the benefits of either of the treatments. All data were collected on a single day.

### 2.6. Statistical Analysis

A total of 190 students were excluded after randomization based on parental consent, which had been obtained before randomization. Although consent status was determined pre-randomization, the post-randomization exclusion of so many students may have weakened the protections that randomization affords the original classroom population. The implications of this exclusion for selection bias and external validity are addressed in the Discussion and Limitations sections. Data from questionnaires were entered into an Excel spreadsheet by research assistants. A double entry was performed to reduce the frequency of data entry errors. New variables were created by taking the difference in the individual items and overall scores for each participant’s KAS and EHI pre- and post-intervention. To ensure that higher scores reflected an increased likelihood of vape abuse, question 10 on the KAS was scored in reverse. Descriptive statistics and graphics for participant demographic characteristics and questionnaire responses were performed before primary analysis to detect errors in data, such as missing data and variables being outside of the plausible range. Eight participants with missing data for entire surveys were removed from the analysis, and those with partial missing data, missing 1 to 2 questions were kept, with the missing questions not included in the study. After this, the database was locked. Initially, we confirmed internal consistency using Cronbach’s alpha to assess the reliability of creating a total score, which was high for the EHI (α = 0.8832 pre-intervention, α = 0.8962 post-intervention) but low for the KAS (α = 0.6219 pre-intervention, α = 0.5382 post-intervention).

The analysis began by describing sample characteristics. Following this, a primary analysis was undertaken to evaluate the success of the intervention. *t*-tests were performed to assess if the intervention group had a significant change between pre- and post-intervention compared to the comparison group on the total EHI score. This was not done for KAS due to its low Cronbach’s alpha score. However, an exploratory analysis was undertaken to specifically explore if the intervention group had a significant change between pre- and post-intervention compared to the comparison group on the individual questions on the KAS and EHI.

We also used *t*-tests to determine if the individual item scores for the first 4 questions on the Video Survey were significantly different between the experimental and comparator groups. A Mann–Whitney U test was used to determine if the two groups responded differently to questions 5 and 6 on the Video Survey. Gender-stratified analyses were also done; however, as most participants were male or female, the models were only stratified into these 2 categories. No multiple comparisons correction was applied to the analysis of individual items as this was considered an exploratory analysis. All analyses, except the gender-stratified analyses and a decision not to analyze internally inconsistent scale scores, adhered to the archived study protocol, which included a sample size calculation and was archived prior to data collection (https://prism.ucalgary.ca/items/51c57a6d-3f1e-41be-9178-0398f47c6087) (accessed on 28 July 2024). Item-level analyses on the KAS, EHI, and Video Survey are therefore exploratory rather than confirmatory. Individually significant item-level results should be interpreted as hypothesis-generating, with the comparison of total EHI scores between groups treated as the primary inferential test.

All analyses were performed on Stata 18 (College Station, TX, USA).

## 3. Results

### 3.1. Sample Descriptives

**Figure 1:**
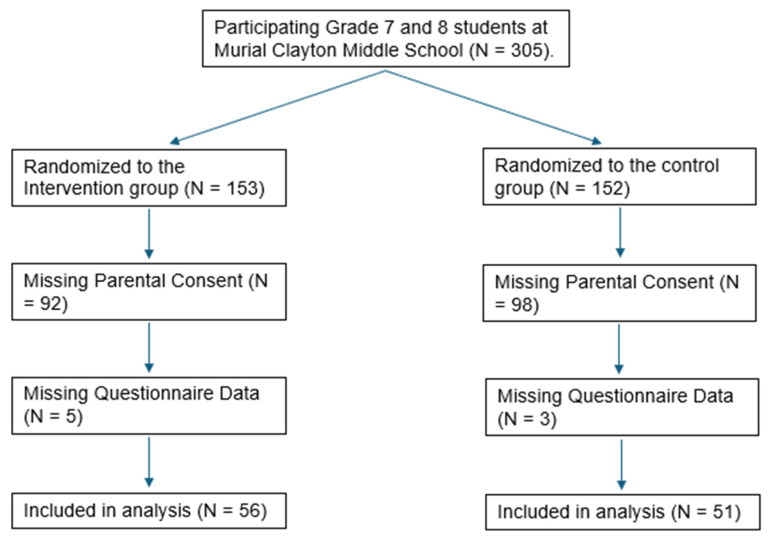
Flow diagram illustrating the selection process of study participants for the evaluation of the ABYA grassroots vaping intervention. Out of 305 initial participants, 190 were excluded due to lack of parental consent. Of the 115 participants included in the study, 8 were further excluded due to incomplete or missing surveys, resulting in 107 participants being included in the final analysis. (Note: although consent forms had been returned to the teacher the research team did not have access to whether the student had consented or not consented).

As shown in Figure 1, 305 Grade 7 and 8 students from Muriel Clayton Middle School were enrolled in the participating classrooms and randomized by class list. One-hundred and fifteen had parental consent forms signed, and of those, 107 had complete questionnaire data. Of the 107 students, 56 were randomized to the intervention group and 51 to the comparison group. The mean age of students in the intervention group was 12.66 (0.66), and 12.75 (0.68) in the comparison group. Most participants had never vaped (97/107); seven had vaped once and three more than once (overall 9.35% ever-vaped). Distribution by group was similar (ABYA 6/56; NIH 4/51). Seven participants had used a vape once (three in the intervention group and four in the comparison group), and three participants had used a vape more than once (all in the intervention group). Therefore, 9.35% of the sample self-reported trying a vape at least once. Demographic characteristics of the sample are outlined in Table 1. There were no negative effects of the intervention reported. The participant flow shown in Figure 1 can be summarized as follows: of the 305 students enrolled in the participating Grade 7 and 8 classrooms, 190 (62.3%) did not have a signed parental consent form returned and were therefore excluded from analysis. An additional 8 of the 115 consented students were excluded after randomization because their booklets contained missing data for one or more entire surveys; partial missing data on individual items were retained, with the missing items omitted from item-level analyses only. The final analytic sample was 107 students.

### 3.2. Questionnaire Results

Because the Cronbach’s alpha for KAS was low, 0.6219 and 0.5382 for the surveys administered before and after respectively, only individual question scores were analyzed for group differences instead of a total score (Table 2). For the question “E-cigarettes are safer than smoking,” the mean decrease of 0.39 (0.80) in the intervention group was significantly larger than the mean increase of 0.02 (0.82) in the comparison group, t(104) = 2.62, *p* = 0.010, with a medium Cohen’s d of 0.509. “Teens use e-cigarettes to get the same buzz they get from tobacco cigarettes” was also significantly different between the intervention and comparison groups, with the intervention group experiencing a mean increase of 0.16 (0.89) and the comparison group a decrease of 0.24 (0.89), t(105) = −2.30, *p* = 0.023, with a Cohen’s d of −0.446. No other significant differences in individual questions on KAS were observed between the two groups.

In the gender-stratified analysis (Supplementary Table S1) no questions were significantly different between the intervention and comparison groups. However, for females, the questions “E-cigarettes are safer than smoking,” “E-cigarettes help people quit using cigarettes,” and “E-cigarette vapor is dangerous to babies and kids” were significantly different, with Cohen’s d values ranging from 0.694 to 0.802. “Teens use e-cigarettes to get the same buzz they get from tobacco cigarettes,” was also significantly different for females between the intervention and comparison groups; however, the effect was negative, with a Cohen’s d of −0.855.

Cronbach’s alpha for EHI was 0.8832 and 0.8962 for before and after respectively; therefore, both total scores and individual items scores were analyzed for differences between the two groups, as planned a priori. The mean score for EHI in the overall sample before the intervention was 32.69 (14.04). Following the intervention, the overall mean EHI score decreased to 27.71 (13.18), with the intervention group experiencing a mean decrease of 9.05 (13.03), which was significantly greater than the decrease observed in the comparison group of 2.06 (7.69), t(105) = 3.34, *p* = 0.001, with a medium effect size of 0.646. Exploratory analyses were conducted to better describe the nature of the change (Table 3). For the question “E-cigarettes are less harmful than cigarettes” the intervention group showed a mean change of 1.21 (2.03), whereas the comparison group had a mean change of 0.26 (1.74). This difference had a Cohen’s d of 0.503 and was statistically significant, t(104) = 2.58, *p* = 0.011. The intervention group had a mean change of 0.84 (1.88) which was significantly larger than the 0.16 (1.17) in the comparison group on the question “E-cigarettes reduce the harmful effects of cigarette smoking” t(104) = 2.21, *p* = 0.030, with a Cohen’s d if 0.429. There was a significant difference between the change seen in the intervention group 1.04 (1.93) and comparison group 0.18 (1.51) on the question “E-cigarettes are healthier than cigarettes”, t(104) = 2.52, *p* = 0.013 with a Cohen’s d of 0.490. The intervention group also showed a significantly greater change on the questions asking “E-cigarette use balances addictions to tobacco and desires to quit” and “E-cigarettes help people quit smoking,” with a difference of 0.76 (0.29) (t(102) = 2.75, *p* = 0.007) and 0.73 (0.36) (t(104) = 2.02, *p* = 0.046) respectively. Both questions also had Cohen’s d values of 0.542 and 0.392 respectively.

In the gender-stratified analysis (Supplementary Table S2) males had significant differences between the intervention and comparison groups for the questions: “E-cigarette use balances addictions to tobacco and desires to quit,” and “E-cigarettes help people quit smoking,” with medium and large effect sizes respectively. The total score on the EHI was not significantly different for males between the two groups, whereas it was significant for females, t(42) = 2.39, *p* = 0.021, with a Cohen’s d effect size of 0.728. “E-cigarettes are healthier than cigarettes” was the only question significantly different between the intervention and comparison groups for females.

On the Video Survey for the question asking about “Increase in knowledge about vaping,” students in the intervention group reported a significantly higher mean score of 3.34 (1.27) compared to the comparator group, which had a mean score of 2.84 (0.95). A *t*-test showed statistical significance between these two means (t(105) = 2.28, *p* = 0.023) with low Cohen’s d of 0.440. Similarly, for the question asking about “Overall enjoyment/entertainment level”, the intervention group again reported a higher mean score of 3.13 (1.11) relative to the other group with a score of 2.33 (0.82), with highly significant differences (t(105) = 4.16, *p* < 0.001). This time the effect size was large at 0.805. No other question was significantly different between the two groups. Video Survey results can be seen in Table 4. As shown in Supplementary Table S3, for the gender-stratified analysis the only question significantly different between the intervention and comparison group was “Overall enjoyment/entertainment level,” with both males and females again having a large effect size over 0.800.

## 4. Discussion

The primary aim of this study was to evaluate the impact of the ABYA grassroots vaping intervention on adolescents’ knowledge, attitudes, and harm perceptions regarding e-cigarette use in Grade 7 and 8 classrooms in Airdrie, Canada. This intervention was compared to a video developed by the NIH.

The results indicated that the ABYA video intervention had a significant impact on increasing the knowledge and altering the perceptions of the students regarding the risks of vaping. The intervention group showed a significant decrease in EHI scores post-intervention, indicating a shift in their understanding of the harm associated with vaping. Significant improvements were also noted in specific EHI survey questions, such as questions related to perceived health benefits, harm reduction, and cessation support of e-cigarettes compared to traditional cigarettes. This emphasizes the intervention’s effectiveness in altering harmful perceptions; however, it is important to understand what may have caused the interventions to impact young people differently. The most important measured factor is how the intervention group rated the ABYA video higher in terms of enjoyment compared to the control group. One factor not considered in the study was that because the ABYA video was designed by non-experts, it presented uncertain information with more confidence, leading to a greater impression on the youth compared to the NIH video. With regard to this, it can be said that the ABYA video exaggerates the risk posed by e-cigarettes, leaving the erroneous impression that e-cigarettes might be more hazardous than cigarettes, while the NIH video can be criticized as being too soft and too uncertain as to such risk.

Regarding the question “E-cigarettes are less harmful than cigarettes” and other items comparing e-cigarettes with cigarettes, we found that students who viewed the intervention video were more likely to rate e-cigarettes unfavorably compared to cigarettes. Since the intervention primarily focused on e-cigarette use and risks and mentioned cigarettes only in the context of nicotine content and addiction potential, participants may have come away with the impression that e-cigarettes are more harmful than cigarettes. While we acknowledge that cigarettes are in fact more harmful than e-cigarettes, we believe the participants’ responses reflect a perception that e-cigarettes are more addictive than cigarettes, which aligns more closely with the information emphasized in the intervention, although research specifically comparing the two is limited [11]. Overall, despite the persistently high prevalence of vaping among adolescents, especially when compared with tobacco cigarette use, it remains critical to educate youth about the harms and risks of e-cigarettes while also reinforcing the risks of traditional tobacco cigarettes [27]. Our intervention focused primarily on vaping, which may explain the responses observed in this study. This highlights the importance of ensuring that future youth-oriented interventions present balanced information on both e-cigarettes and traditional cigarettes, since adolescents may encounter both. In the local context of Airdrie, however, existing school and community programs had already emphasized the dangers of cigarette smoking while giving little attention to vaping. This gap helps explain why the ABYA may have overemphasized e-cigarettes’ risks compared to traditional cigarettes. In designing youth-facing materials, balanced relative-risk communication is critical, particularly in settings where established tobacco-focused interventions are not prevalent. A potential benefit of grassroots approaches lies in their capacity to assess local contexts and tailor messaging to address identified gaps in existing prevention efforts, thereby enhancing the appropriateness and potential impact of the intervention. Although the ABYA video may have introduced a risk of overstating the harms of vaping relative to cigarettes, the present data cannot determine whether the educational benefits outweighed that risk or whether relative-risk misunderstanding could affect future nicotine-related choices. Future youth-facing materials should preserve the engaging peer-led format while explicitly communicating that combustible cigarettes remain more harmful than e-cigarettes.

This finding raises an important ethical and public health consideration. Although the ABYA video produced larger gains in perceived risk of e-cigarettes, it appears to have done so partly by understating the substantial harm differential between e-cigarettes and combustible tobacco. Adolescents who internalize the belief that e-cigarettes are as harmful as, or more harmful than, traditional cigarettes may be less likely to consider e-cigarettes as a harm-reduction option if they later become smokers, and may underestimate the considerable risks of combustible tobacco. Conversely, messaging that emphasizes the relative safety of e-cigarettes too strongly risks reducing perceived risk of vaping itself. Future grassroots interventions should retain the engagement advantages of youth-developed messaging while explicitly situating e-cigarettes within the broader nicotine-product harm continuum so that the resulting risk perceptions are both accurate and protective.

To our knowledge, this is one of the first randomized controlled evaluations of grassroots, youth-led vaping intervention. While past research has consistently shown that traditional school-based e-cigarette prevention programs have limited effectiveness in reducing initiation (e.g., Williams et al. [19]), few studies have directly tested whether youth-led approaches can outperform expert-developed materials. Our findings align with broader public health literature demonstrating that grassroots or peer-led interventions can enhance credibility, relatability, and engagement among adolescents, leading to stronger shifts in health perceptions and message engagement [28]. This suggests that youth-led approaches may help overcome the shortcomings identified in recent evaluations of e-cigarette prevention programs [20].

As was outlined in the analysis plan, due to the low Cronbach’s alpha, the overall score on the KAS was not assessed, and instead individual questions were assessed. On the KAS, specific questions such as “E-cigarettes are safer than smoking” showed significant improvements in the intervention group, whereas for another question the intervention group performed worse than the comparison group (“Teens use e-cigarettes to get the same buzz they get from tobacco cigarettes”). This highlights the effectiveness of the ABYA video in certain aspects of knowledge and attitudes towards vaping while also showing potential limitations. Ultimately, however, the results from the KAS survey should not be taken into consideration due to a lack of internal consistency based on Cronbach’s alpha test. The low reliability of the KAS scale in our sample likely reflects two main issues: (1) the heterogeneous nature of the items, which measured both factual knowledge (e.g., chemical content) and attitudinal beliefs (e.g., perceived safety), and (2) limited variance in responses, as most participants had never used a vape and tended to respond uniformly. Together, these factors reduced internal consistency and limited the interpretability of the composite score.

Three further methodological considerations should weigh on the interpretation of these findings. First, although random assignment was completed before consent status was known to the research team, 190 of the 305 enrolled students were excluded because their parents had not returned a signed consent form. This was mainly attributed to a short window of 3 days between students receiving the consent form to when the trial began. Students whose parents granted consent may still however differ systematically from those whose parents did not on dimensions such as parental engagement with school, household communication about substance use, or socio-demographic background, and the analytic sample may therefore not be fully representative of the broader Grade 7 and 8 population in this school or elsewhere. Because exclusions reduced the sample from 305 enrolled students to 107 complete cases, randomization should be interpreted as supporting comparison within this selected analytic sample rather than entirely eliminating selection bias from the original eligible population. Second, the great majority of participants (90.7%) reported never using a vape. The intervention’s effects on adolescents who are current or former e-cigarette users may differ in important ways, particularly given that perceived-risk shifts produced by messaging may not translate into the same shifts among users whose attitudes have been shaped by personal experience. Third, the trial was conducted in a single school in Airdrie within an existing local context emphasizing tobacco prevention; the generalizability of these findings to schools with different demographic compositions, different baseline programming, or different regional norms around vaping cannot be assumed and will require multi-site replication.

Additionally, survey results from the Video Survey indicated that students in the intervention group reported a significantly higher increase in knowledge and enjoyment from the ABYA video compared to the NIH control video. This suggests that the grassroots nature and community-led design of the ABYA video were more engaging and impactful for the students.

The gender-stratified analysis highlighted notable differences in how the intervention impacted perceptions of e-cigarettes. For females, significant improvements were observed in questions about the risks of e-cigarettes, including their safety, ability to help quit smoking, and dangers to children. In contrast, males showed significant changes in perceptions of e-cigarettes as tools for managing addiction and quitting smoking. While the overall EHI score was significantly different between the two groups for females, it was not for males. Notably, both genders reported high enjoyment of the intervention, underscoring its broad appeal. These results suggest the intervention was effective but impacted males and females differently.

Implications for practice and policy are notable. First, schools may benefit from incorporating youth-driven, peer-created materials into their health curricula to enhance relevance and relatability. Second, public health agencies could partner with youth councils or community groups to co-develop interventions, thereby ensuring that messaging is grounded in adolescent perspectives. Finally, policymakers should recognize grassroots initiatives as viable complements to traditional prevention strategies, particularly in addressing emerging health issues like vaping where credibility with young audiences is essential.

Ultimately, this study has demonstrated that youth-led, grassroots interventions can outperform traditional expert-driven methods in improving adolescents’ vaping knowledge and altering harmful perceptions. Through involving peers and community members in both the design and delivery of the messages, these interventions address critiques of conventional programs that may lack developmental appropriateness and real-world relatability. Consistent with prior findings [19,20], our study highlights the strong impact of youth-informed, co-created materials in changing vaping related attitudes and harm perceptions.

### Limitations and Future Directions

The relatively small sample size limits the generalizability of the findings. The specific demographic and geographic focus of the study may not be entirely representative of the broader adolescent population. Future research should explore the impact of similar grassroots interventions on larger and more diverse populations (i.e., different schools and municipalities outside Airdrie). A key limitation for this research was that limited demographic information was collected, and it is important that future work analyzes the role of demographics more thoroughly. Another limitation is that only a small subset of participants reported current vaping, which reduces the generalizability of findings to more frequent or established vapers; the impact of the intervention on habitual vaping behaviors may not be fully captured in this study. Longitudinal studies are recommended to assess the long-term impact of grassroots interventions on vaping behaviors. It would be beneficial to evaluate the sustainability of changes in knowledge, attitudes, and perceptions over periods of weeks or months post-intervention. Moreover, due to the low reliability of the KAS instrument in our sample, conclusions regarding changes in knowledge and attitudes should be interpreted with caution. Future research should employ more robust and psychometrically sound measures to better capture these constructs. Although we measured entertainment, we did not measure perceived credibility, and this may be an important factor future studies should consider when assessing the efficacy of grassroots-led efforts. Lastly, as the ABYA video was completely led by the community, some information in the video did not consider nuanced scientific discord in the field, thus potentially presenting a biased view.

Furthermore, one limitation of this study is the use of certain questions in our questionnaires that compare e-cigarettes to traditional cigarettes. These questionnaires were not originally developed or validated specifically for this study, and our intervention and control videos primarily focused on the risks of e-cigarettes rather than cigarettes. As a result, changes in participants’ responses to these comparison questions before and after the intervention may not fully reflect the most up-to-date understanding of cigarette versus e-cigarette risks.

Several specific limitations should be highlighted. First, the post-randomization exclusion of 190 students whose parents did not return a signed consent form raises the possibility of selection bias, as consenting and non-consenting families may differ on characteristics relevant to adolescent vaping. Second, no behavioral outcomes such as vaping initiation, frequency, or intention were measured and as such the conclusions of this study are therefore restricted to changes in knowledge, attitudes, and harm perceptions. Any extension to behavior should be regarded as speculative until tested in trials with behavioral endpoints. Third, the KAS instrument showed low internal consistency in this sample (Cronbach’s alpha = 0.54 to 0.62); although this was anticipated in the analysis plan, it limits the inference that can be drawn from KAS-based outcomes, and item-level findings on the KAS are exploratory rather than confirmatory. Fourth, neither the KAS nor the EHI has been formally validated in Canadian Grade 7 and 8 students, and the psychometric properties observed here may not transfer to other adolescent populations. Fifth, no correction was applied for the multiple item-level comparisons performed across the KAS, EHI, and Video Survey, so individually significant items should be treated as hypothesis-generating. Finally, the trial took place in a single school within a community that had pre-existing tobacco-prevention programming, which limits external validity to other settings.

There is potential for applying the grassroots intervention model to other public health issues beyond vaping, such as drug or alcohol abuse. Successful grassroots initiatives could also be scaled up for broader community and national implementation, leveraging the power of peer influence and community involvement.

## 5. Conclusions

In this randomized trial conducted in a single Alberta middle school, a youth-developed grassroots vaping video produced a significantly greater shift in adolescents’ harm perceptions of e-cigarettes than an expert-developed comparison video, with a medium effect size on total EHI scores, and was rated significantly higher by students for both overall enjoyment and perceived increase in knowledge about vaping. The study did not measure vaping behavior or behavioral intention, and the low internal consistency of the KAS limits inferences that can be drawn about changes in knowledge and attitudes. Within these constraints, the findings provide initial evidence that grassroots, youth-driven interventions can outperform conventional expert-developed materials in altering adolescent e-cigarette harm perceptions, and they support further multi-site evaluation of this model with longitudinal behavioral endpoints and built-in safeguards for accurate relative-risk framing.

## Figures and Tables

**Table 1:** Demographic statistics of the study participants.

Characteristics	Category	Overall (107)	Group A (56)	Group B (51)
Number		107	56 (52.3%)	51 (47.7%)
Age				
	12	45 (42.1%)	25 (44.6%)	20 (39.2%)
	13	49 (45.8%)	25 (44.6%)	24 (47.1%)
	14	13 (12.1%)	6 (10.7%)	7 (13.7%)
Grade				
	7	55 (51.4%)	31 (55.4%)	24 (47.1%)
	8	51 (47.7%)	25 (44.6%)	26 (51.0%)
	Other	1 (0.9%)	0 (0%)	1 (2.0%)
Gender				
	Male	54 (50.5%)	26 (46.4%)	28 (54.9%)
	Female	44 (41.1%)	25 (44.6%)	19 (37.3%)
	Non-Binary	1 (0.9%)	1 (1.8%)	0 (0%)
	Agender	1 (0.9%)	1 (1.8%)	0 (0%)
	Prefer Not To Say	5 (4.7%)	2 (3.6%)	3 (5.9%)
	Other	2 (1.9%)	1 (1.8%)	1 (2.0%)
Vaping Status				
	Never	97 (90.7%)	50 (89.3%)	47 (92.2%)
	Once	7 (6.5%)	3 (5.4%)	4 (7.8%)
	More Than Once	3 (2.8%)	3 (5.4%)	0 (0%)

**Table 2:** Knowledge and attitudes regarding e-cigarette ingredients, safety, and addictive properties before and after scores by group.

Question	Overall Mean (SD)		Group A Mean (SD)		Group B Mean (SD)		*p*-Value	Cohen’s d
	Before	After	Before	After	Before	After		
Smoke from e-cigarettes is just water	1.42 (0.61)	1.27 (0.59)	1.50 (0.69)	1.34 (0.69)	1.33 (0.52)	1.20 (0.45)	0.860	0.034
E-cigarettes don’t contain tar	2.08 (0.76)	1.86 (0.77)	2.05 (0.75)	1.84 (0.76)	2.10 (0.79)	1.88 (0.79)	0.992	−0.002
E-cigarettes aren’t addictive	1.20 (0.56)	1.21 (0.64)	1.25 (0.58)	1.21 (0.56)	1.14 (0.53)	1.20 (0.72)	0.521	0.125
E-cigarettes aren’t a tobacco product	2.02 (0.92)	1.87 (0.94)	2.02 (0.94)	1.95 (1.00)	2.02 (0.88)	1.78 (0.86)	0.345	−0.184
E-cigarettes don’t produce smoke	1.46 (0.74)	1.49 (0.73)	1.45 (0.74)	1.54 (0.83)	1.47 (0.76)	1.43 (0.61)	0.420	−0.157
Using e-cigarettes feels cleaner than smoking	2.18 (0.93)	1.98 (0.92)	2.11 (0.91)	1.89 (0.87)	2.26 (0.96)	2.09 (0.97)	0.908	0.023
E-cigarettes are safer than smoking	1.90 (0.90)	1.70 (0.82)	2.02 (0.88)	1.63 (0.80)	1.76 (0.92)	1.78 (0.84)	0.010	0.509
Teens use e-cigarettes to get the same buzz they get from tobacco cigarettes	2.80 (0.84)	2.78 (0.94)	2.66 (0.82)	2.82 (0.96)	2.96 (0.85)	2.73 (0.94)	0.023	−0.446
E-cigarettes help people quit using cigarettes	1.89 (0.86)	2.01 (0.91)	1.96 (0.87)	1.96 (0.85)	1.80 (0.85)	2.06 (0.97)	0.157	0.276
E-cigarette vapor is dangerous to babies and kids	1.41 (0.87)	1.66 (1.12)	1.41 (0.87)	1.52 (1.03)	1.41 (0.88)	1.82 (1.20)	0.169	0.268

*p*-values and Cohen’s d values are for the mean difference in before and after scores between Group A (intervention) and Group B (comparison).

**Table 3:** E-cigarette Harm Perception and Reduction items before and after scores by group.

Question	Overall Mean (SD)		Group A Mean (SD)		Group B Mean (SD)		*p*-Value	Cohen’s d
	Before	After	Before	After	Before	After		
E-cigarettes are less harmful than cigarettes.	2.91 (1.81)	2.14 (1.66)	3.18 (1.77)	1.96 (1.48)	2.60 (1.84)	2.33 (1.83)	0.011	0.503
E-cigarettes reduce the harmful effects of cigarette smoking.	2.50 (1.68)	1.99 (1.51)	2.86 (1.80)	2.02 (1.61)	2.12 (1.46)	1.96 (1.40)	0.029	0.429
E-cigarettes cut down on the harmful effects of secondhand smoke.	2.54 (1.54)	2.05 (1.21)	2.71 (1.55)	2.00 (1.13)	2.35 (1.53)	2.10 (1.30)	0.144	0.286
E-cigarettes provide a safer way to get nicotine.	2.41 (1.50)	1.93 (1.36)	2.73 (1.53)	2.04 (1.41)	2.06 (1.39)	1.80 (1.30)	0.148	0.282
E-cigarettes are lower in tar or carbon monoxide than cigarettes.	3.19 (1.59)	2.31 (1.48)	3.38 (1.56)	2.36 (1.63)	2.98 (1.62)	2.25 (1.29)	0.384	0.169
E-cigarettes make smoking safer.	2.21 (1.46)	1.89 (1.44)	2.48 (1.62)	1.89 (1.46)	1.90 (1.16)	1.88 (1.42)	0.068	0.360
E-cigarettes are healthier than cigarettes.	2.61 (1.84)	1.98 (1.59)	2.84 (1.95)	1.80 (1.49)	2.35 (1.68)	2.18 (1.67)	0.013	0.490
E-cigarettes improve breathing and reduce coughing.	1.57 (1.20)	1.44 (0.92)	1.80 (1.43)	1.52 (1.01)	1.31 (0.81)	1.35 (0.82)	0.168	0.269
E-cigarettes do not release toxins into the environment.	1.82 (1.50)	1.73 (1.23)	1.89 (1.45)	1.88 (1.31)	1.75 (1.57)	1.57 (1.14)	0.639	−0.091
E-cigarettes help improve sense of smell and taste.	1.75 (1.42)	1.59 (1.08)	1.89 (1.60)	1.63 (1.12)	1.59 (1.19)	1.55 (1.05)	0.368	0.175
E-cigarettes are a good compromise for people trying to stop cigarettes.	2.63 (1.77)	2.41 (1.78)	2.95 (1.90)	2.45 (1.72)	2.28 (1.57)	2.37 (1.87)	0.061	0.368
E-cigarette use balances addictions to tobacco and desires to quit. *	2.34 (1.50)	2.01 (1.44)	2.75 (1.62)	1.98 (1.37)	1.88 (1.22)	2.04 (1.54)	0.007	0.542
E-cigarettes are less addictive than cigarettes.	2.22 (1.62)	1.72 (1.24)	2.45 (1.50)	1.84 (1.36)	1.96 (1.71)	1.59 (1.10)	0.617	0.099
E-cigarettes help people quit smoking.	2.37 (1.73)	2.18 (1.68)	2.64 (1.72)	2.11 (1.57)	2.06 (1.71)	2.25 (1.81)	0.046	0.392

*p*-values and Cohen’s d values for the mean difference in before and after scores between Group A (intervention) and Group B (comparison). * Note: This question is referring to the belief that vaping can help manage nicotine dependence during quit attempts.

**Table 4:** Video survey results by group.

Question	Overall Mean (SD)	Group A Mean (SD)	Group B Mean (SD)	*p*-Value	Cohen’s d
Increase in Knowledge	3.10 (1.15)	3.34 (1.27)	2.84 (0.95)	0.023	0.440
Usefulness for Other Students	3.63 (1.15)	3.55 (122)	3.71 (1.08)	0.498	−0.132
Overall Enjoyment	2.75 (1.06)	3.13 (1.11)	2.33 (0.82)	<0.001	0.805
Knowledge About Risks	4.27 (0.89)	4.23 (0.87)	4.31 (0.91)	0.636	−0.092
Effect on Interest in Vaping					
Made me less likely; *n* (%)	76 (71.03%)	41 (73.21)	35 (68.63%)	0.603	
Did not impact my choice; *n* (%)	31 (28.97%)	15 (26.79)	16 (31.37%)		
Difficulty of Material					
Too Easy; *n* (%)	22 (20.56%)	8 (14.29%)	14 (27.45%)	0.088	
Just Right; *n* (%)	82 (76.64%)	46 (82.14%)	36 (70.59%)		
Too Difficult; *n* (%)	3 (2.80%)	2 (3.57%)	1 (1.96%)		

The first 4 questions were answered on a Likert scale 1 through 5 corresponding to very poor, poor, satisfactory, good, excellent. Significant differences in mean scores on the first 4 questions were assessed using *t*-tests, whereas for the last 2 questions Mann–Whitney U-Tests were used.

## Data Availability

The data that support the findings of this study are available from the corresponding author upon reasonable request.

## Supplementary Materials

The following supporting information can be downloaded at: https://www.mdpi.com/article/10.3390/ijerph23060789/s1, Survey S1: Video Survey, Survey S2: Knowledge and Attitudes Regarding E-cigarette Ingredients, Safety, and Addictive Properties survey, Survey S3: The E-cigarette Harm Perception and Reduction survey, Survey S4: Personal Information Questionnaire, Table S1: Mean difference between before and after scores on the Knowledge and attitudes regarding e-cigarette ingredients, safety, and addictive properties by group stratified by gender. Table S2: Mean difference between before and after scores on the E-cigarette Harm Perception and Reduction Items stratified by sex. Table S3: Video Survey Results by Group stratified by sex. The first 4 questions were answered on a Likert scale 1 through 5 corresponding to very poor, poor, satisfactory, good, excellent. Significant differences in mean scores on the first 4 questions were assessed using t-tests, whereas for the last 2 questions Mann-Whitney U-Tests were used.

## Abbreviations

The following abbreviations are used in this manuscript:

ABYA	Airdrie Board of Youth Affairs
EHI	E-cigarette Harm Perception and Reduction (survey)
KAS	Knowledge and Attitudes Regarding E-cigarette Ingredients, Safety, and Addictive Properties (survey)
NIH	National Institute of Health
REB	Research Ethics Board
TCPS	Tri-Council Policy Statement
U of C	University of Calgary
