# Supplementary material for: Community-Driven Grassroots Intervention on Adolescent Vaping Attitudes, Harm Perceptions, and Knowledge: Randomized Controlled Trial
Source: Int J Environ Res Public Health. 2026 Jun 11;23(6):789. doi: 10.3390/ijerph23060789 (PMC13299536; doi:10.3390/ijerph23060789)
Supplement: Supplementary file 1 [file ijerph-23-00789-s001.zip › Vaping Intervention Study Supplementary Tables.pdf]

Table S1. Mean difference between before and after scores on the Knowledge and attitudes regarding e-cigarette ingredients, safety, and addictive properties by group stratified by gender.

Questions	Male				Female			
	Group A Mean (SD)	Group B Mean (SD)	p-value	Cohen's d	Group A Mean (SD)	Group B Mean (SD)	p-value	Cohen's d
Smoke from e-cigarettes is just water	0.04 (0.92)	0.14 (0.36)	0.578	-0.152	0.20 (0.82)	0.11 (0.57)	0.668	0.132
E-cigarettes don't contain tar	0.28 (1.02)	0.48 (0.70)	0.408	-0.232	0.04 (1.10)	-0.11 (0.99)	0.653	0.138
E-cigarettes aren't addictive	0.00 (0.85)	-0.04 (0.64)	0.861	0.048	0.04 (0.84)	-0.11 (0.90)	0.575	0.175
E-cigarettes aren't a tobacco product	0.19 (0.69)	0.44 (1.05)	0.309	-0.282	0.04 (0.84)	-0.05 (1.03)	0.744	0.1
E-cigarettes don't produce smoke	-0.15 (0.78)	0.07 (0.81)	0.306	-0.282	0.04 (0.98)	-0.16 (0.50)	0.426	0.245
Using e-cigarettes feels cleaner than smoking	0.08 (0.93)	0.19 (0.90)	0.651	-0.126	0.20 (0.91)	0.18 (1.33)	0.946	0.021
E-cigarettes are safer than smoking	0.23 (0.71)	0.04 (0.65)	0.305	0.285	0.36 (0.76)	-0.21 (0.79)	0.019	0.741
Teens use e-cigarettes to get the same buzz they get from tobacco cigarettes	-0.15 (0.78)	0.11 (0.83)	0.242	-0.322	-0.32 (0.85)	0.47 (1.02)	0.007	-0.855
E-cigarettes help people quit using cigarettes	-0.27 (1.15)	-0.18 (0.90)	0.748	-0.088	0.28 (0.74)	-0.37 (0.90)	0.012	0.802

E-cigarette vapor is dangerous to babies and kids	-0.04 (1.18)	-0.18 (0.90)	0.625	0.134	0.04 (1.10)	-0.74 (1.15)	0.028	0.694

Table S2. Mean difference between before and after scores on the E-cigarette Harm Perception and Reduction Items stratified by sex.

Questions	Male				Female			
	Group A Mean (SD)	Group B Mean (SD)	p-value	Cohen's d	Group A Mean (SD)	Group B Mean (SD)	p-value	Cohen's d
E-cigarettes are less harmful than cigarettes.	1.15 (1.93)	0.41 (1.80)	0.152	0.4	1.12 (2.11)	-0.05 (1.78)	0.058	0.594
E-cigarettes reduce the harmful effects of cigarette smoking.	0.65 (1.90)	0.00 (0.62)	0.095	0.467	0.84 (1.60)	0.32 (1.73)	0.305	0.316
E-cigarettes cut down on the harmful effects of secondhand smoke.	0.40 (1.22)	0.50 (1.75)	0.813	-0.065	0.76 (1.54)	-0.05 (1.39)	0.078	0.551
E-cigarettes provide a safer way to get nicotine.	0.58 (1.65)	0.39 (1.40)	0.66	0.121	0.96 (1.51)	0.00 (2.00)	0.077	0.552
E-cigarettes are lower in tar or carbon monoxide than cigarettes.	0.73 (1.80)	0.89 (1.71)	0.736	-0.092	1.12 (1.48)	0.47 (1.87)	0.207	0.39
E-cigarettes make smoking safer.	0.42 (1.90)	-0.07 (1.54)	0.3	0.288	0.68 (1.55)	0.17 (1.82)	0.325	0.308
E-cigarettes are healthier than cigarettes.	0.69 (1.81)	0.57 (1.43)	0.785	0.075	1.28 (1.95)	-0.39 (1.65)	0.005	0.912
E-cigarettes improve breathing and reduce coughing.	0.00 (1.52)	-0.04 (0.74)	0.912	0.03	0.20 (0.96)	-0.05 (0.71)	0.339	0.294

E-cigarettes do not release toxins into the environment.	0.04 (1.64)	-0.04 (2.15)	0.888	0.039	0.16 (1.34)	0.42 (1.46)	0.543	-0.187
E-cigarettes help improve sense of smell and taste.	-0.19 (0.94)	0.00 (1.05)	0.483	-0.192	0.48 (1.53)	0.16 (1.07)	0.438	0.238
E-cigarettes are a good compromise for people trying to stop cigarettes.	0.50 (1.77)	0.00 (1.22)	0.229	0.331	0.28 (2.09)	-0.33 (1.61)	0.304	0.322
E-cigarette use balances addictions to tobacco and desires to quit.	1.00 (1.74)	0.11 (1.09)	0.03	0.615	0.56 (1.94)	-0.29 (1.10)	0.109	0.516
E-cigarettes are less addictive than cigarettes.	0.19 (1.81)	0.43 (1.83)	0.636	-0.13	0.84 (1.86)	0.47 (1.23)	0.478	0.225
E-cigarettes help people quit smoking.	0.88 (1.40)	-0.32 (1.59)	0.005	0.805	0.00 (2.63)	0.17 (1.72)	0.816	-0.073

Table S3. Video Survey Results by Group stratified by sex. The first 4 questions were answered on a Likert scale 1 through 5 corresponding to very poor, poor, satisfactory, good, excellent. Significant differences in mean scores on the first 4 questions were assessed using t-tests, whereas for the last 2 questions Mann-Whitney U-Tests were used.

**Question**	**Male**				**Female**			
	**Group A Mean (SD)**	**Group B Mean (SD)**	**p-value**	**Cohen's d**	**Group A Mean (SD)**	**Group B Mean (SD)**	**p-value**	**Cohen's d**
Increase in Knowledge	3.31 (1.29)	2.82 (0.94)	0.118	0.451	3.20 (1.29)	2.89 (0.88)	0.381	0.265
Usefulness for Other Students	3.38 (1.27)	3.86 (1.01)	0.134	-0.426	3.64 (1.19)	3.53 (1.22)	0.757	0.096
Overall Enjoyment	3.19 (1.06)	2.50 (0.69)	0.006	0.819	3.12 (1.17)	2.21 (0.79)	0.005	0.87
Knowledge About Risks	4.15 (0.78)	4.46 (0.58)	0.102	-0.454	4.28 (0.98)	4.11 (1.24)	0.605	0.156
Effect on Interest in Vaping								
Made me less likely; *n (%)*	19 (73.08)	20 (71.43)	0.894		20 (80.00)	13 (68.42)	0.385	
Did not impact my choice; *n (%)*	7 (26.92)	8 (28.57)			5 (20.00)	6 (31.58)		
Difficulty of Material								
Too Easy; *n (%)*	5 (19.23)	10 (35.71)			3 (12.00)	3 (15.79)		
Just Right; *n (%)*	20 (76.92)	17 (60.71)	0.212		22 (88.00)	16 (84.21)	0.720	
Too Difficult; *n (%)*	1 (3.85)	1 (3.57)			0 (0.00)	0 (0.00)		

Supplementary Information:

Intervention: https://www.youtube.com/watch?v=SC4hZk8OgrI

Comparaison : https://www.youtube.com/watch?v=IN7iCZJ3H6w
