# Supplementary material for: Community-Driven Grassroots Intervention on Adolescent Vaping Attitudes, Harm Perceptions, and Knowledge: Randomized Controlled Trial
Source: Int J Environ Res Public Health. 2026 Jun 11;23(6):789. doi: 10.3390/ijerph23060789 (PMC13299536; doi:10.3390/ijerph23060789)
Supplement: Supplementary file 1 [file ijerph-23-00789-s001.zip › Survey S1-Video Survey.pdf]

Video Survey

Please rate the Vaping Video in the following areas according to your experience from (Very Poor (1), Poor, Satisfactory, Good, Excellent (5))

1. *The increase in your knowledge about vaping?* ① ② ③ ④ ⑤

2. *How useful do you think it is for other students in your grade?* ① ② ③ ④ ⑤

3. *Overall enjoyment/entertainment level?* ① ② ③ ④ ⑤

4. *After completing this, how would you rate your knowledge about the risks of vaping?* ① ② ③ ④ ⑤

5. Did this activity affect your interest in vaping?

- ☐ This activity made me less likely to try vaping in the future
- ☐ This activity did not impact my choice

6. Did you feel that the health-related material was too easy, just right, or too difficult to understand?

- ☐ Too easy
- ☐ Just right
- ☐ Too difficult

7. What was your favorite part of this activity and why?

8. What parts of this activity did you not like? What could we change to improve it?

Knowledge and attitudes regarding e-cigarette ingredients, safety, and addictive properties

Please read each statement carefully and indicate your level of agreement in the box that corresponds with your opinion. The scale ranges from 4 (Strongly Agree) to 1 (Strongly Disagree).

Your responses are completely confidential and will be used for research purposes only. There are no right or wrong answers; we are interested in your honest opinions.

Statement	Strongly Agree (4)	Agree (3)	Disagree (2)	Strongly Disagree (1)
**Smoke from e-cigarettes is just water**				
**E-cigarettes don't contain tar**				
**E-cigarettes aren't addictive**				
**E-cigarettes aren't a tobacco product**				
**E-cigarettes don't produce smoke**				
**Using e-cigarettes feels cleaner than smoking**				
**E-cigarettes are safer than smoking**				
**Teens use e-cigarettes to get the same buzz they get from tobacco cigarettes**				
**E-cigarettes help people quit using cigarettes**				
**E-cigarette vapor is dangerous to babies and kids**				

Thank you for participating in our survey. Please return the completed survey in the envelope provided. We appreciate your time and effort in helping us with this research.

Ethics ID: REB23-1437
This study has been approved by the University of Calgary Conjoint Health Research Ethics Board

E-cigarette Harm Perception and Reduction Items

Please read each statement and indicate the number that best reflects your opinion.
1 means "Do not agree" and 7 means "agree".

		Do not agree						agree
**#**	**Statement**	**1**	**2**	**3**	**4**	**5**	**6**	**7**
**Harm Reduction**								
**1**	E-cigarettes are less harmful than cigarettes.							
**2**	E-cigarettes reduce the harmful effects of cigarette smoking.							
**3**	E-cigarettes cut down on the harmful effects of secondhand smoke.							
**4**	E-cigarettes provide a safer way to get nicotine.							
**5**	E-cigarettes are lower in tar or carbon monoxide than cigarettes.							
**6**	E-cigarettes make smoking safer.							
**7**	E-cigarettes are healthier than cigarettes.							
**Health Benefits**								
**8**	E-cigarettes improve breathing and reduce coughing.							
**9**	E-cigarettes do not release toxins into the environment.							
**10**	E-cigarettes help improve sense of smell and taste.							
**Smoking Cessation**								
**11**	E-cigarettes are a good compromise for people trying to stop cigarettes.							
**12**	E-cigarette use balances addictions to tobacco and desires to quit.							
**13**	E-cigarettes are less addictive than cigarettes.							
**14**	E-cigarettes help people quit smoking.							

Please return the survey in the envelope provided. Thank you for your participation!

Ethics ID: REB23-1437
This study has been approved by the University of Calgary Conjoint Health Research Ethics Board
