# Supplementary material for: Community-Driven Grassroots Intervention on Adolescent Vaping Attitudes, Harm Perceptions, and Knowledge: Randomized Controlled Trial
Source: Int J Environ Res Public Health. 2026 Jun 11;23(6):789. doi: 10.3390/ijerph23060789 (PMC13299536; doi:10.3390/ijerph23060789)
Supplement: Supplementary file 1 [file ijerph-23-00789-s001.zip › Survey S2-Knowledge and attitudes regarding e-cigarette ingredients, safety, and addictive properties.pdf]

Knowledge and attitudes regarding e-cigarette ingredients, safety, and addictive properties

Please read each statement carefully and indicate your level of agreement in the box that corresponds with your opinion. The scale ranges from 4 (Strongly Agree) to 1 (Strongly Disagree).

Your responses are completely confidential and will be used for research purposes only. There are no right or wrong answers; we are interested in your honest opinions.

Statement	Strongly Agree (4)	Agree (3)	Disagree (2)	Strongly Disagree (1)
**Smoke from e-cigarettes is just water**				
**E-cigarettes don't contain tar**				
**E-cigarettes aren't addictive**				
**E-cigarettes aren't a tobacco product**				
**E-cigarettes don't produce smoke**				
**Using e-cigarettes feels cleaner than smoking**				
**E-cigarettes are safer than smoking**				
**Teens use e-cigarettes to get the same buzz they get from tobacco cigarettes**				
**E-cigarettes help people quit using cigarettes**				
**E-cigarette vapor is dangerous to babies and kids**				

Thank you for participating in our survey. Please return the completed survey in the envelope provided. We appreciate your time and effort in helping us with this research.

UNIVERSITY OF CALGARY

Ethics ID: REB23-1437
This study has been approved by the University of Calgary Conjoint Health Research Ethics Board
