# Supplementary material for: Community-Driven Grassroots Intervention on Adolescent Vaping Attitudes, Harm Perceptions, and Knowledge: Randomized Controlled Trial
Source: Int J Environ Res Public Health. 2026 Jun 11;23(6):789. doi: 10.3390/ijerph23060789 (PMC13299536; doi:10.3390/ijerph23060789)
Supplement: Supplementary file 1 [file ijerph-23-00789-s001.zip › Survey S3-E-cigarette Harm Perception and Reduction Items.docx.pdf]

E-cigarette Harm Perception and Reduction Items

Please read each statement and indicate the number that best reflects your opinion.
1 means "Do not agree" and 7 means "agree".

		Do not agree						agree
**#**	**Statement**	**1**	**2**	**3**	**4**	**5**	**6**	**7**
**Harm Reduction**								
**1**	E-cigarettes are less harmful than cigarettes.							
**2**	E-cigarettes reduce the harmful effects of cigarette smoking.							
**3**	E-cigarettes cut down on the harmful effects of secondhand smoke.							
**4**	E-cigarettes provide a safer way to get nicotine.							
**5**	E-cigarettes are lower in tar or carbon monoxide than cigarettes.							
**6**	E-cigarettes make smoking safer.							
**7**	E-cigarettes are healthier than cigarettes.							
**Health Benefits**								
**8**	E-cigarettes improve breathing and reduce coughing.							
**9**	E-cigarettes do not release toxins into the environment.							
**10**	E-cigarettes help improve sense of smell and taste.							
**Smoking Cessation**								
**11**	E-cigarettes are a good compromise for people trying to stop cigarettes.							
**12**	E-cigarette use balances addictions to tobacco and desires to quit.							
**13**	E-cigarettes are less addictive than cigarettes.							
**14**	E-cigarettes help people quit smoking.							

Please return the survey in the envelope provided. Thank you for your participation!

UNIVERSITY OF CALGARY

Ethics ID: REB23-1437
This study has been approved by the University of Calgary Conjoint Health Research Ethics Board
