# Supplementary material for: Community-Driven Grassroots Intervention on Adolescent Vaping Attitudes, Harm Perceptions, and Knowledge: Randomized Controlled Trial
Source: Int J Environ Res Public Health. 2026 Jun 11;23(6):789. doi: 10.3390/ijerph23060789 (PMC13299536; doi:10.3390/ijerph23060789)
Supplement: Supplementary file 1 [file ijerph-23-00789-s001.zip › Survey S4-Personal Information Questionnaire.pdf]

Personal Information Questionnaire

Please answer the following questions to help us understand our demographic better. Your responses will remain confidential.

Gender: Which of the following best describes your gender identity? Please select the option(s) that best represent you or provide your preferred term.

- ☐ Male
- ☐ Female
- ☐ Non-binary
- ☐ Transgender
- ☐ Agender
- ☐ Genderqueer
- ☐ Prefer not to say
- ☐ Other (Please specify): __________

Age: Please tick the box next to your age. If your age is not listed, select 'Other' and specify.

- ☐ 11 years old
- ☐ 12 years old
- ☐ 13 years old
- ☐ 14 years old
- ☐ 15 years old
- ☐ 16 years old
- ☐ Other (Please specify): __________

Grade: What is your current educational grade? If your grade is not listed, select 'Other' and specify.

- ☐ Grade 7
- ☐ Grade 8
- ☐ Other (Please specify): __________

Have you ever used a vape/electronic cigarette? Please remember your answers are confidential and your honesty is appreciated.

- ☐ Never
- ☐ Once
- ☐ More than once

UNIVERSITY OF CALGARY

Ethics ID: REB23-1437
This study has been approved by the University of Calgary Conjoint Health Research Ethics Board
